# Autoimmune hepatitis triggered by nitrofurantoin: a case series

**DOI:** 10.1186/1752-1947-4-311

**Published:** 2010-09-23

**Authors:** Sally Appleyard, Ruma Saraswati, David A Gorard

**Affiliations:** 1 Wycombe Hospital, Queen Alexandra Road, High Wycombe, Bucks HP11 2TT, UK

## Abstract

**Introduction:**

Drugs can occasionally trigger the onset of autoimmune liver disease.

**Case presentation:**

Three Caucasian women (aged 65, 42 and 74 years old) who were receiving long-term nitrofurantoin as prophylaxis against recurrent urinary tract infections developed hepatitic liver disease. Serological auto-antibody profiles and liver histology appearances were consistent with autoimmune hepatitis. Two of the patients presented with jaundice, and one required a prolonged hospital admission for liver failure. In all three patients nitrofurantoin was withdrawn, and long-term immunosuppressive therapy with prednisolone and azathioprine or mycophenolate was given. The patients responded well, with liver biochemistry returning to normal within a few months.

**Conclusions:**

Although nitrofurantoin rarely causes autoimmune hepatitis, this antimicrobial is increasingly used as long-term prophylaxis against recurrent urinary tract infection. General practitioners and urologists who prescribe long-term nitrofurantoin therapy should be aware of this adverse effect.

## Introduction

Autoimmune liver disease is a not uncommon cause of chronic hepatitis in women. Although autoimmune destruction usually occurs without an identifiable trigger, some drugs including methyldopa [1] and minocycline [2] are associated with autoimmune liver disease. The antimicrobial nitrofurantoin has also rarely been implicated in autoimmune hepatitis. We report here three patients with hepatitis in whom nitrofurantoin was felt to be etiologically important.

### Case 1

A 65-year-old Caucasian retired police woman developed non-specific symptoms of abdominal discomfort, nausea and lethargy. She later became jaundiced, with dark urine and pale stools. She had a past history of irritable bowel syndrome, chronic lumbar back pain and recurrent urinary tract infection. Her drug history included mebeverine 135 mg thrice daily, dosulepin 25 mg daily, lansoprazole 30 mg daily, nitrofurantoin 50 mg daily for six years, occasional paracetamol and inhaled salbutamol, and intermittent fluconazole for groin candidiasis. She took no herbal remedies or unprescribed supplements and had not drunk any alcohol for more than 20 years.

On examination she was jaundiced, had a tender liver edge and mild ankle edema, but no other peripheral stigmata of liver disease. Initial blood tests revealed deranged liver function with bilirubin 649 μmol/L (normal range 4-21), alanine transaminase 1322 U/L (10-35), and alkaline phosphatase 227 U/L (40-150). Albumin was low at 28 g/L (35-50), but her coagulation screen was normal. Full blood count, electrolytes and renal function were normal, with a platelet count of 196 × 10^9^/L.

Serological testing for hepatitis A, hepatitis B and hepatitis C was negative. Liver ultrasound revealed unremarkable appearances of the liver and biliary system, with no evidence of gallstones or biliary dilatation. Immunological tests demonstrated positive smooth muscle antibody and positive anti-nuclear antibody homogenous staining pattern at a titre of 1 in 320. There was an accompanying hyper-gammaglobulinemia with elevated IgG at 21.8 g/L (normal range 5.8-14) and normal IgA and IgG levels. Double stranded DNA antibodies, anti-mitochondrial antibodies and liver/kidney microsomal antibodies were not detected.

**Figure 1:**
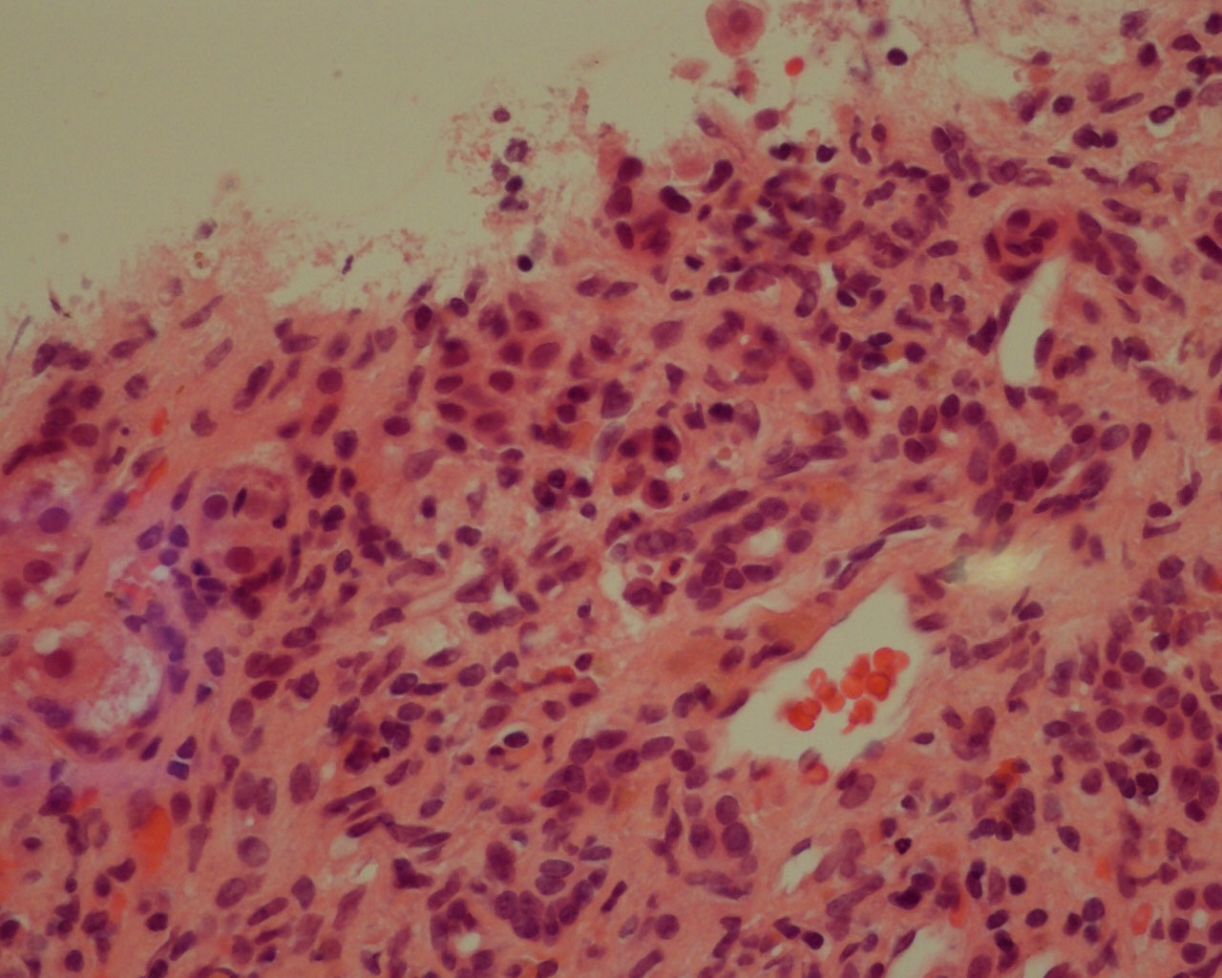
**Liver biopsy in Case 1: portal tract inflammation with moderate to severe chronic inflammatory cell infiltrate composed of lymphocytes with a few plasma cells (hematoxylin and eosin × 40)**.

The positive anti-nuclear and smooth muscle antibody results were strongly suggestive of autoimmune hepatitis. Liver biopsy was performed and this showed fibrous expansion of portal tracts but without evidence of established necrosis. A chronic inflammatory cell infiltrate with interface hepatitis and piecemeal necrosis was evident in portal areas. The histological features were consistent with autoimmune hepatitis (Figure 1).

Initial management consisted of oral prednisolone 25 mg daily and bone protection using alendronate once weekly. Within a few weeks she had a good clinical response with resolution of jaundice and lethargy. With the exception of nitrofurantoin which was suspected of having triggered her illness, all her regular medications which had all been temporarily stopped during her investigations, were restarted. Liver biochemistry improved with bilirubin dropping from 649 to 62 μmol/L, and alanine transaminase dropping from 1322 U/L to 118 U/L after only one month of prednisolone. The prednisolone dose was subsequently reduced and azathioprine was added as a steroid sparing agent. After two years she remains well with normal liver biochemistry while no longer taking prednisolone and solely taking azathioprine 150 mg daily for her chronic hepatitis.

### Case 2

A 42-year-old Caucasian woman, who worked as a nurse, was found to have an elevated alanine transaminase concentration of 469 U/L (10-35), during investigation of sub-fertility. The rest of her liver biochemistry including albumin, and her coagulation screen were normal. She had no symptoms or signs of liver disease and her past medical history was notable only for recurrent urinary tract infections and previous miscarriages. Her only medications were folic acid and nitrofurantoin 50 mg daily for two years. There was no history of excess alcohol intake nor ingestion of herbal remedies or supplements. Clinical examination was unremarkable.

**Figure 2:**
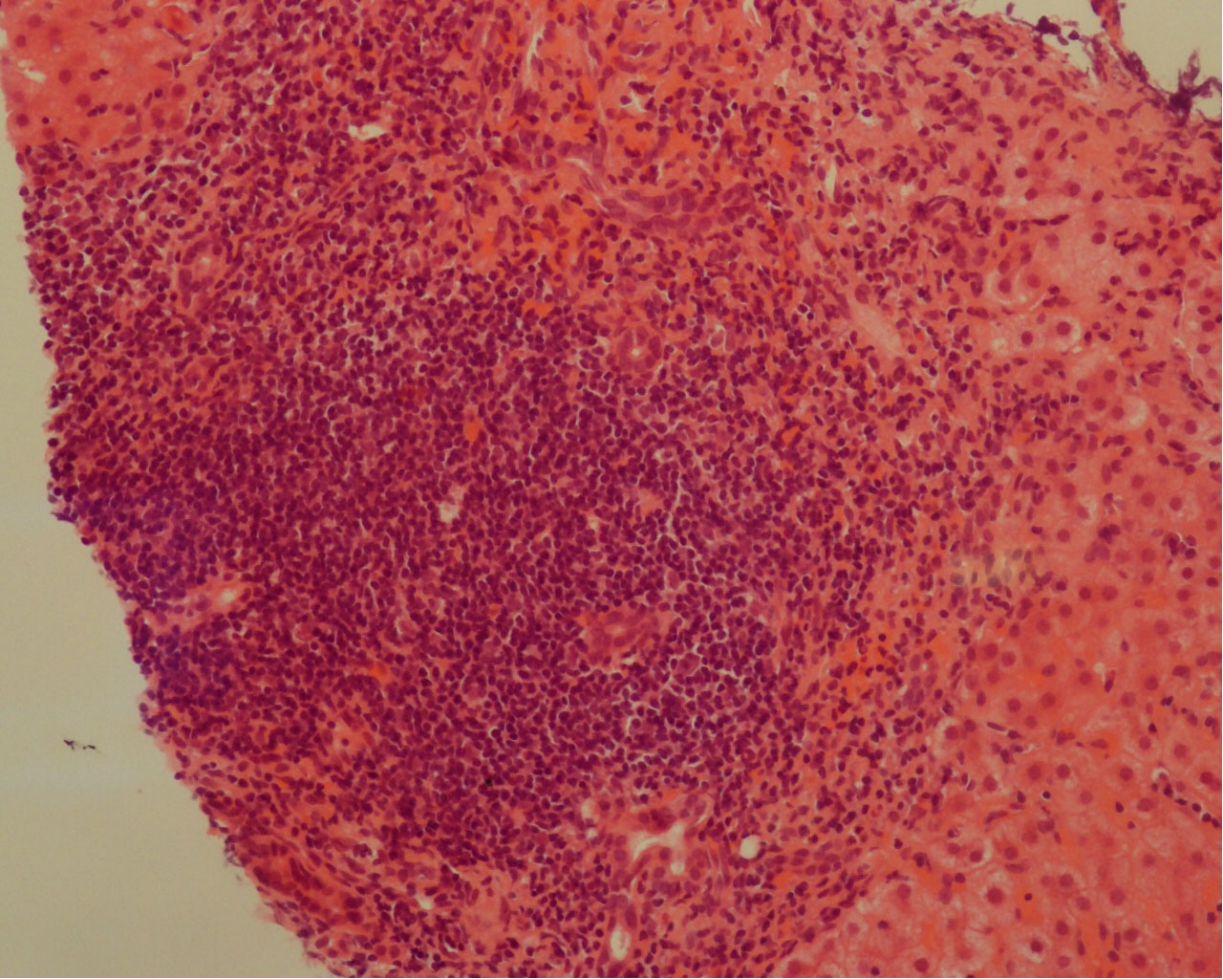
**Liver biopsy in Case 2: severe chronic inflammation within portal tracts (hematoxylin and eosin × 20)**.

Further investigations revealed a very abnormal autoimmune screen including positive anti-nuclear antibodies at a titre of 1 in 640, smooth muscle antibodies at a titre of 1 in 320 and anti-mitochondrial antibodies at a titre of 1 in 320. Anti-Rho, extractable nuclear antibody and rheumatoid factor were also positive. IgG levels were elevated at 22.8 g/L, while IgA and IgM levels were normal. Serological testing for chronic hepatitis viruses was negative and the serum ferritin was 300 μg/L. Liver biopsy showed marked chronic inflammation within the portal tracts and extensive fibrosis and some features of cirrhosis (Figure 2).

She was treated with oral steroids at an initial dose of 40 mg of prednisolone daily. Nitrofurantoin was stopped. Since she was trying to become pregnant a bone-protecting bisphosphonate was withheld because of teratogenicity risks. Her liver biochemistry improved rapidly after a few weeks of corticosteroid medication. Plans to introduce azathioprine have been postponed while she tries to conceive. Two years after diagnosis she remains well with entirely normal liver biochemistry while taking prednisolone 7.5 mg daily.

### Case 3

A 74-year-old Caucasian woman with a previous history of cholecystectomy and left nephrectomy for renal carcinoma four years earlier, presented with jaundice, nausea, dark urine and pale stools. Her only medication was nitrofurantoin 100 mg daily for two years as prophylaxis against urinary tract infections. There was no history of excess alcohol intake. An ultrasound scan showed an irregular liver contour, her previous cholecystectomy and no evidence of any biliary obstruction. Clinical examination revealed palmar erythema, a palpable liver edge, cholecystectomy and nephrectomy scars.

**Figure 3:**
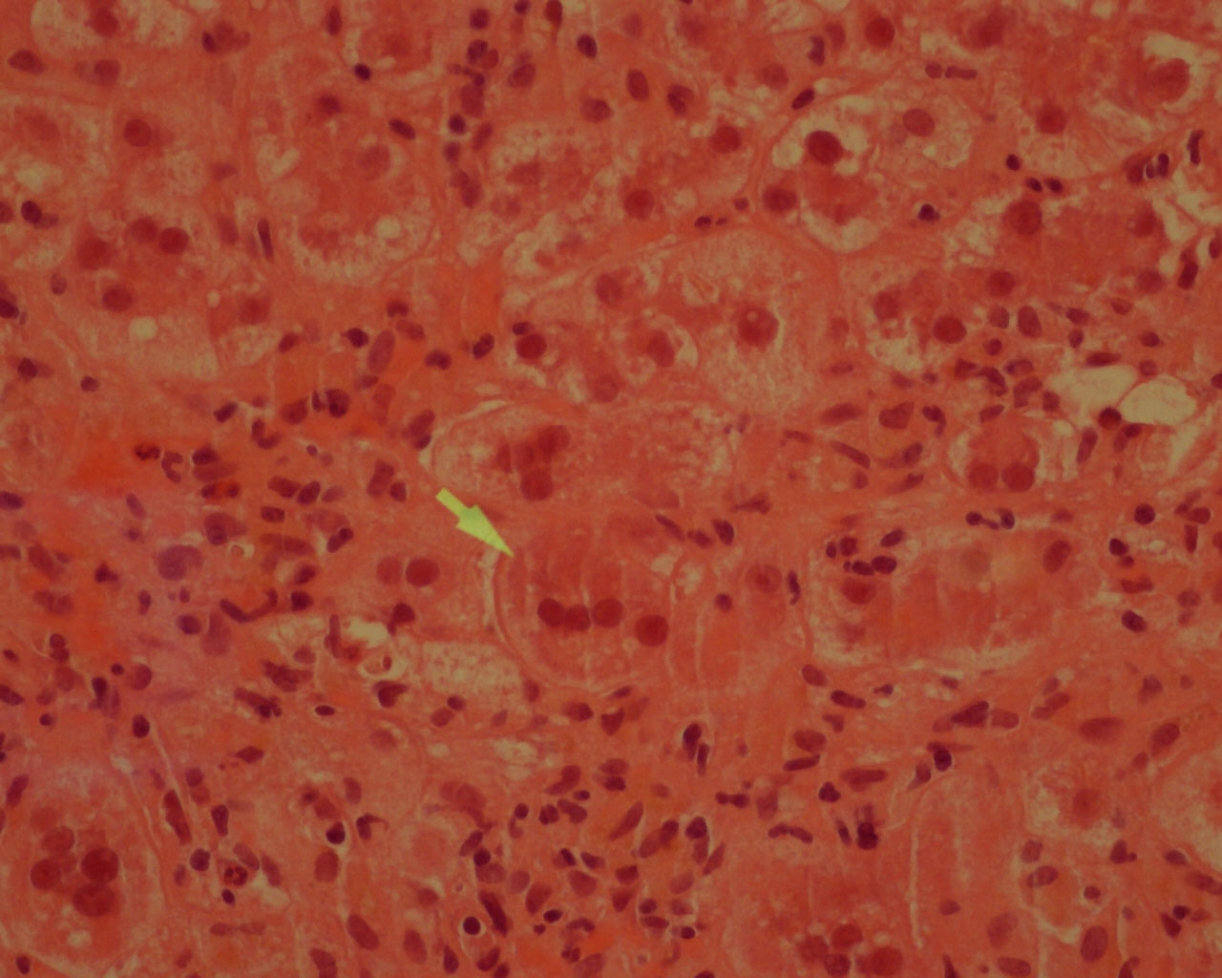
**Liver biopsy in Case 3: syncitial hepatocyte giant cell (arrowed) with surrounding severe hydropic degenerative change of hepatocytes and numerous neutrophils (hematoxylin and eosin × 40)**.

Blood tests on admission showed unremarkable full blood count (FCB), although mean corpuscular volume (MCV) was 100, bilirubin 394 μmol/L, alanine aminotransferase (ALT) 1423 U/L, alkaline phosphatase 207 U/L. Albumin was 29 g/L, international normalized ratio (INR) 1.2. A computed tomography (CT) scan of the abdomen showed no focal lesion within the liver and no evidence of any biliary dilatation. Serological tests were negative for hepatitis A, hepatitis B, hepatitis C, hepatitis E, cytomegalovirus, Epstein-Barr virus and human immunodeficiency virus (HIV). Immunoglobulin levels showed high IgG at 22.3 g/L and IgA slightly elevated at 4.4 g/L. Anti-nuclear antibody was strongly positive at 1:640, but smooth muscle antibody and liver/kidney microsomal antibodies were negative as was the rest of the auto-antibody screen including serological markers for systemic lupus erythematosus. Tissue transglutaminase antibody was negative. A liver biopsy showed striking lobular inflammation, confluent and bridging necrosis. In addition there were some syncytial giant cells. There was minimal portal inflammation and minimal plasma cells. Features were consistent with an acute hepatitis with an autoimmune component and some features of a syncytial giant cell hepatitis (Figure 3).

Nitrofurantoin was stopped. Prednisolone 30 mg daily was commenced, but failed to improve her clinical or biochemical picture. The dose was increased to 50 mg daily, and she remained hospitalized as she became more unwell with bilirubin reaching 430 μmol/L and albumin dropping to16 g/L. Mycophenolate 1 g twice daily was added. Over a period of four months her jaundice subsided and at seven months after initial presentation her liver biochemistry had returned to normal, while taking prednisolone 10 mg daily and mycophenolate 1 g twice daily.

## Discussion

Three cases of hepatitis in women who were taking long-term nitrofurantoin as prophylaxis against recurrent urinary tract infection are described. In all three cases the serology, liver histology and clinical response to immunosuppressive drugs were consistent with autoimmune hepatitis.

Nitrofurantoin is widely used for both acute and chronic management of urinary tract infections. It is cheap and effective, with a low incidence of resistance in common urinary pathogens; it is also safe in pregnancy [3]. Nitrofurantoin has been linked to autoimmune hepatitis, but in view of the rarity of the association, almost all reports of the association have been single case reports or small series [4-9]. Further information has been obtained from national adverse drug reaction monitoring agencies in the Netherlands [10] and Denmark [11] and it has been estimated that the incidence of nitrofurantoin-induced hepatic injury is low at about three cases in 1,000,000 [12].

Liver injury secondary to nitrofurantoin has been reported after both acute and chronic exposure and the patterns of hepatotoxity are different. Case reports have often reported heterogeneous patients [7], including both acute and chronic nitrofurantoin associated liver injury. Acute injury, which may be via a hypersensitivity type reaction, tends to occur within weeks of drug introduction [8]. Chronic hepatitis is associated with long-term antimicrobial prophylaxis. It occurs after at least six months treatment in 85% of cases [10], and in many cases treatment has been ongoing for years before problems present. Anti-nuclear antibodies and anti-smooth muscle antibodies are often present in the chronic form of hepatitis [6] and the liver histology resembles that of 'idiopathic' autoimmune hepatitis.

When hepatotoxicity is a consequence of direct drug injury, drug withdrawal alone may lead to liver recovery. However, the chronic liver disease associated with nitrofurantoin is likely to be mediated via an immunoallergenic route. Evidence for this includes the increase incidence in women, presence of auto-antibodies and histological appearances. Risk factors for nitrofurantoin-triggered liver injury have been postulated and these include advanced age (possibly related to reduced creatinine clearance), female sex - in common with many autoimmune diseases, and length of exposure. In addition to nitrofurantoin, other drugs such as methyldopa, dihydralazine and minocycline are also implicated in inducing immune-mediated liver disease. In drug-induced autoimmune liver disease it is believed that drug metabolites act as haptens and covalently bind to macromolecular protein carriers. In those genetically predisposed, the altered proteins are perceived as foreign and consequently provoke an immunological attack on normal hepatocellular components [13].

Nitrofurantoin-induced hepatitis is commonly associated with hyper-gammaglobulinemia [14] and with anti-nuclear and anti-smooth muscle antibodies [6]. Our first patient demonstrated both these antibodies, our second patient had a widely positive autoimmune screen and our third patient had anti-nuclear antibodies alone. This heterogeneity of auto-antibody production is also seen in patients with autoimmune hepatitis and no known drug trigger. The auto-antibody status of the three patients prior to nitrofurantoin exposure is unknown. None of these three patients had any record of liver auto-antibodies being checked before the onset of their liver disease.

The natural history of nitrofurantoin-induced hepatitis is variable. There are certainly patients who have progressed to fatal liver disease or transplant [7-9], but it is also likely that many cases are sub-clinical and remain undiagnosed.

Management of nitrofurantoin-triggered hepatitis depends firstly on recognition of the causal relationship. Prognosis is generally good if nitrofurantoin is withdrawn early, and sometimes simply withdrawing nitrofurantoin is sufficient to reverse the hepatic damage [7]. However, corticosteroids are useful in patients who had persistently abnormal liver biochemistry despite withdrawal of nitrofurantoin. In the cases reported here, our patients had been on nitrofurantoin for considerable periods of time and since the biochemical, serological and histopathological data supported an immunologically-mediated disease process, simple withdrawal of nitrofurantoin without the use of immunosuppressive drugs was deemed insufficient. Thus all three of our patients were treated with prednisolone, resulting in a rapid improvement in the first two cases, but a rather slow improvement in the third. Immunosuppression with corticosteroids may fail to improve patients diagnosed at a very advanced or fulminant stage of disease. Such fatal outcomes have occurred when nitrofurantoin either has been continued after liver dysfunction was identified, or has been reintroduced after liver damage was first detected [6,7].

Future re-challenge with nitrofurantoin should be avoided since the recurrent liver dysfunction may be persistent and even fatal. In one case repeat administration of nitrofurantoin 17 years after the initial exposure led to recurrent liver disease [15].

Hepatitis is not the only potentially serious side-effect of nitrofurantoin; it has also been associated with pulmonary, hematological and neurological complications. Nitrofurantoin induced pulmonary disease and liver injury can occur together and it may well be that these share a common autoimmune mechanism [16,17]. The pulmonary disease is often the predominant feature and so those patients with interstitial pneumonitis should have liver function tests to check for occult hepatitis.

## Conclusions

Nitrofurantoin is widely used in the long term prophylaxis of recurrent urinary tract infection. Serious hepatic injury remains rare, but recognition of harm from nitrofurantoin is crucial since early withdrawal of the drug has been shown to result in improved prognosis. Consideration should be given to monitoring liver function tests during long term nitrofurantoin therapy.

## Competing interests

The authors declare that they have no competing interests.

## Consent

Written informed consent was obtained from the three patients for publication of these case reports and any accompanying images. A copy of the written consent is available for review by the Editor-in-Chief of this journal.

## Authors' contributions

SA collated patient data regarding the liver disease, and prepared the first manuscript draft. RS performed the histological examination of the liver biopsies. DG redrafted the manuscript and acted as corresponding author. All authors read and approved the final manuscript.
